# Supplementary material for: Host and microbial factors influence bacterial colonization of the honey bee gut
Source: ISME J. 2026 May 18;20(1):wrag127. doi: 10.1093/ismejo/wrag127 (PMC13293248; doi:10.1093/ismejo/wrag127)
Supplement: Supplementary_material_wrag127 [file supplementary_material_wrag127.zip › SUPPLEMENTARY MATERIAL.pdf]

SUPPLEMENTARY MATERIAL

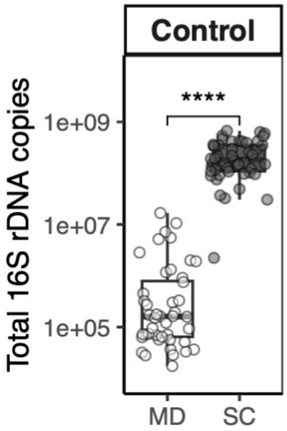

Figure S1: **Total bacterial load in control groups (MD and SC) seven days post-inoculation.** Total 16S rRNA gene copies per gut were quantified by qPCR using universal bacterial primers, in microbiota depleted (MD) bees and native synthetic community (SC) colonized bees. Mann–Whitney test, *n* =48. Significant label: *P* <=0.0001:****.

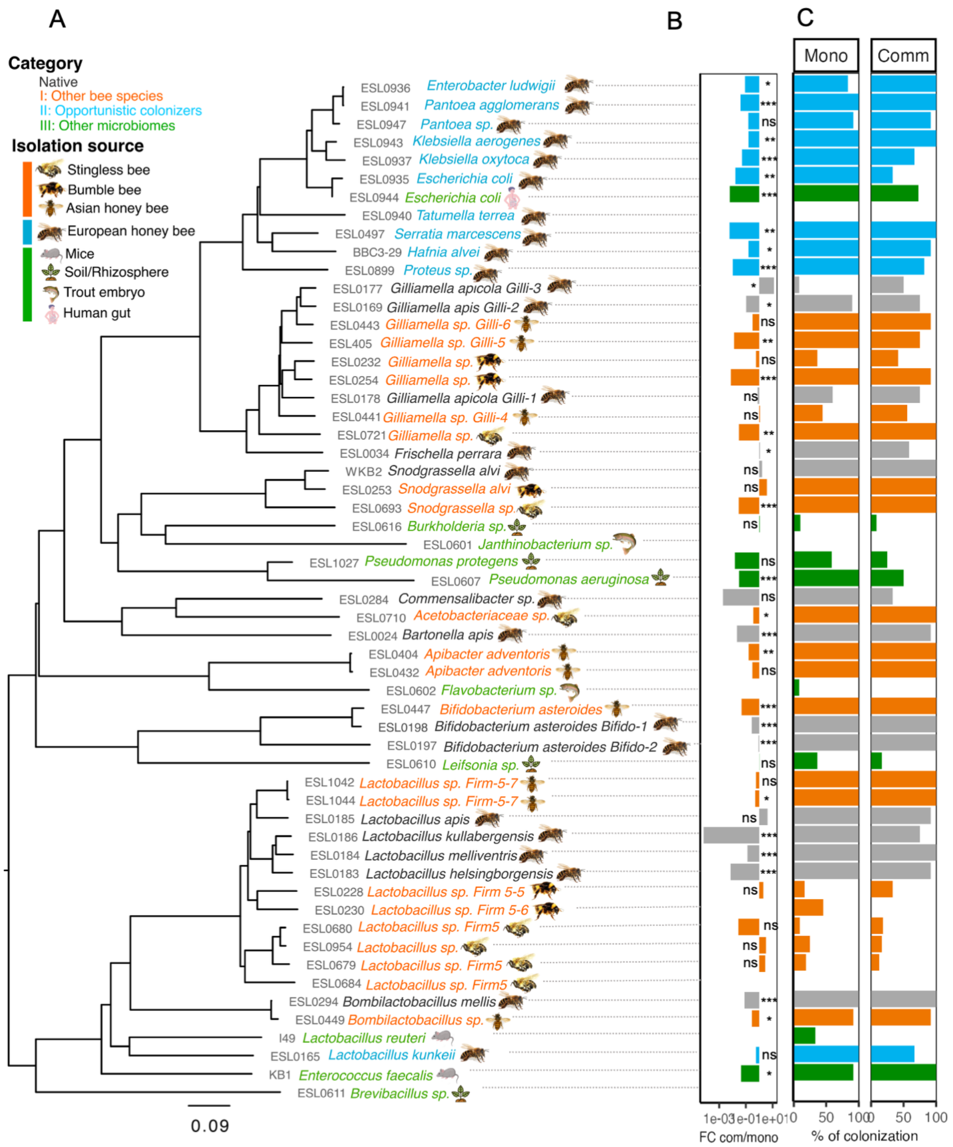
A
B
C
Category
Native
I: Other bee species
II: Opportunistic colonizers
III: Other microbiomes
Isolation source
Stingless bee
Bumble bee
Asian honey bee
European honey bee
Mice
Soil/Rhizosphere
Trout embryo
Human gut
Mono
Comm
0.09
FC com/mono
% of colonization
ESL0936 Enterobacter ludwigii
ESL0941 Pantoea agglomerans
ESL0947 Pantoea sp.
ESL0943 Klebsiella aerogenes
ESL0937 Klebsiella oxytoca
ESL0935 Escherichia coli
ESL0944 Escherichia coli
ESL0940 Tatumella terrea
ESL0497 Serratia marcescens
BBC3-29 Hafnia alvei
ESL0899 Proteus sp.
ESL0177 Gilliamella apicola Gilli-3
ESL0169 Gilliamella apis Gilli-2
ESL0443 Gilliamella sp. Gilli-6
ESL405 Gilliamella sp. Gilli-5
ESL0232 Gilliamella sp.
ESL0254 Gilliamella sp.
ESL0178 Gilliamella apicola Gilli-1
ESL0441 Gilliamella sp. Gilli-4
ESL0721 Gilliamella sp.
ESL0034 Frischella perrara
WKB2 Snodgrassella alvi
ESL0253 Snodgrassella alvi
ESL0693 Snodgrassella sp.
ESL0616 Burkholderia sp.
ESL0601 Janthinobacterium sp.
ESL1027 Pseudomonas protegens
ESL0607 Pseudomonas aeruginosa
ESL0284 Commensalibacter sp.
ESL0710 Acetobacteriaceae sp.
ESL0024 Bartonella apis
ESL0404 Apibacter adventoris
ESL0432 Apibacter adventoris
ESL0602 Flavobacterium sp.
ESL0447 Bifidobacterium asteroides
ESL0198 Bifidobacterium asteroides Bifido-1
ESL0197 Bifidobacterium asteroides Bifido-2
ESL0610 Leifsonia sp.
ESL1042 Lactobacillus sp. Firm-5-7
ESL1044 Lactobacillus sp. Firm-5-7
ESL0185 Lactobacillus apis
ESL0186 Lactobacillus kullabergensis
ESL0184 Lactobacillus melliventris
ESL0183 Lactobacillus helsingborgensis
ESL0228 Lactobacillus sp. Firm 5-5
ESL0230 Lactobacillus sp. Firm 5-6
ESL0680 Lactobacillus sp. Firm5
ESL0954 Lactobacillus sp.
ESL0679 Lactobacillus sp. Firm5
ESL0684 Lactobacillus sp. Firm5
ESL0294 Bombilactobacillus mellis
ESL0449 Bombilactobacillus sp.
I49 Lactobacillus reuteri
ESL0165 Lactobacillus kunkeii
KB1 Enterococcus faecalis
ESL0611 Brevibacillus sp.

7

2

Figure S2. Bacterial colonization success of the honey bee gut, seven days post-inoculation, in monocolonization or in the presence of the native synthetic community (SC). **A** Phylogenetic tree of the 56 bacterial species and strains tested for colonization of the honey bee gut. The tree was inferred with OrthoFinder using the STAG algorithm[51]. **B.** Difference in abundance (expressed in log10 fold change) of each strain between monocolonization and in the presence of the native community. Mono and Comm data were obtained from independent bees; the fold-change shown connects the same strain measured under the two conditions. Wilcoxon rank-sum test. The *P* values were adjusted using FDR method. Significance labels shown as *P*<=0.05:*, *P*=<=0.01:**, *P*<=0.001***, ns: not significant. **C.** Percent of colonization success for each strain in monocolonization (Mono) and in the presence of the native community (Comm) based on qPCR absolute abundance calculated as genome equivalents. The gut of a bee was considered colonized when the abundance was higher than in the initial inoculum (see material and methods). All colonization experiments were conducted with n=12 bees, obtained from three independent experimental replicates conducted with bees from different bee hives.

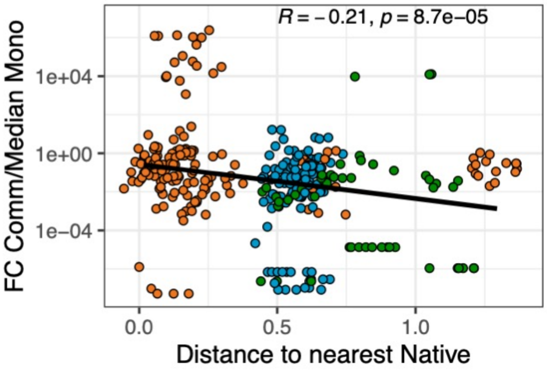

Figure S3: Fold change (FC) in the load of non-native strains in monocolonization relative to colonization in community, plotted against the phylogenetic distance to their nearest native strain. Phylogenetic distances are the horizontal distances in the phylogenetic tree (Figure 1A). *R* depicts Spearman correlation coefficient. Point colors indicate ecological category (as defined in Fig. 1A).

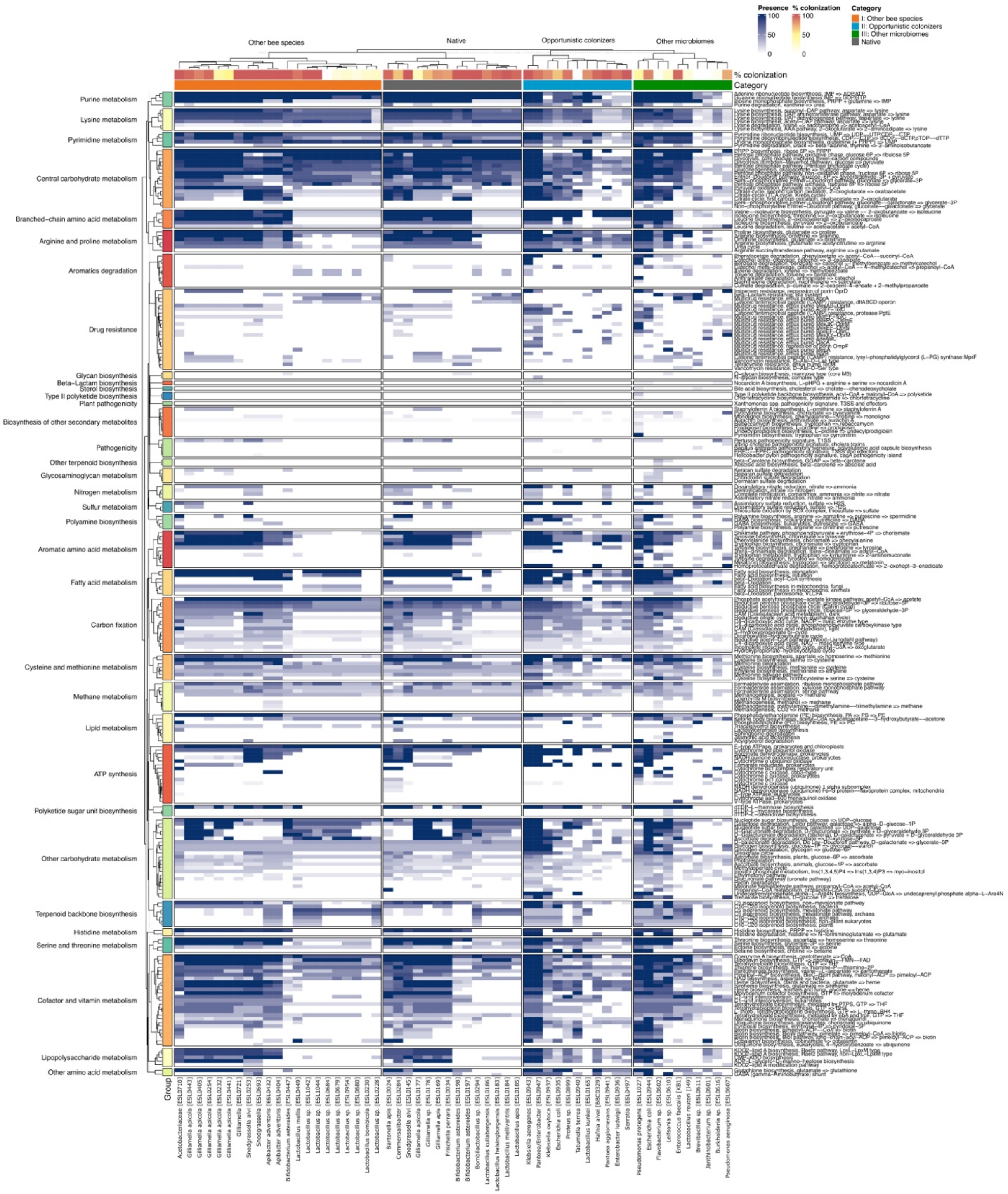

Figure S4: The presence and completeness of KEGG modules in the genomes of individual strains. Heatmap of percent of completeness for each strain. Supplementary File S4.

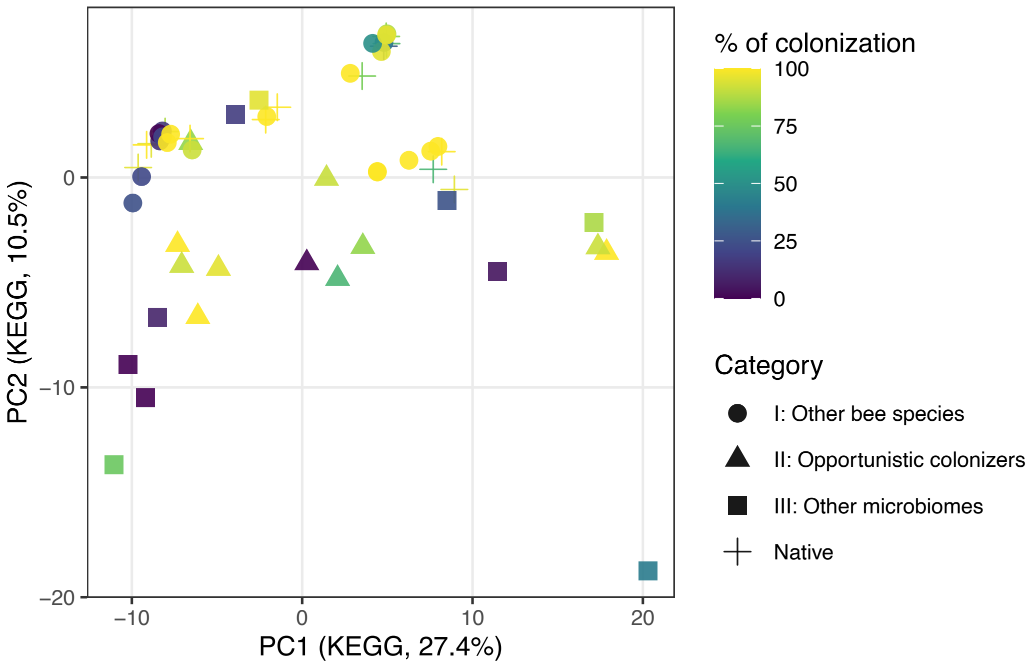

Figure S5: PCA of KEGG module completeness across strains. Principal component analysis was performed on KEGG module completeness profiles. Each point is a strain, coloured by its colonization efficiency in monocolonization. The shape indicates the isolation category.

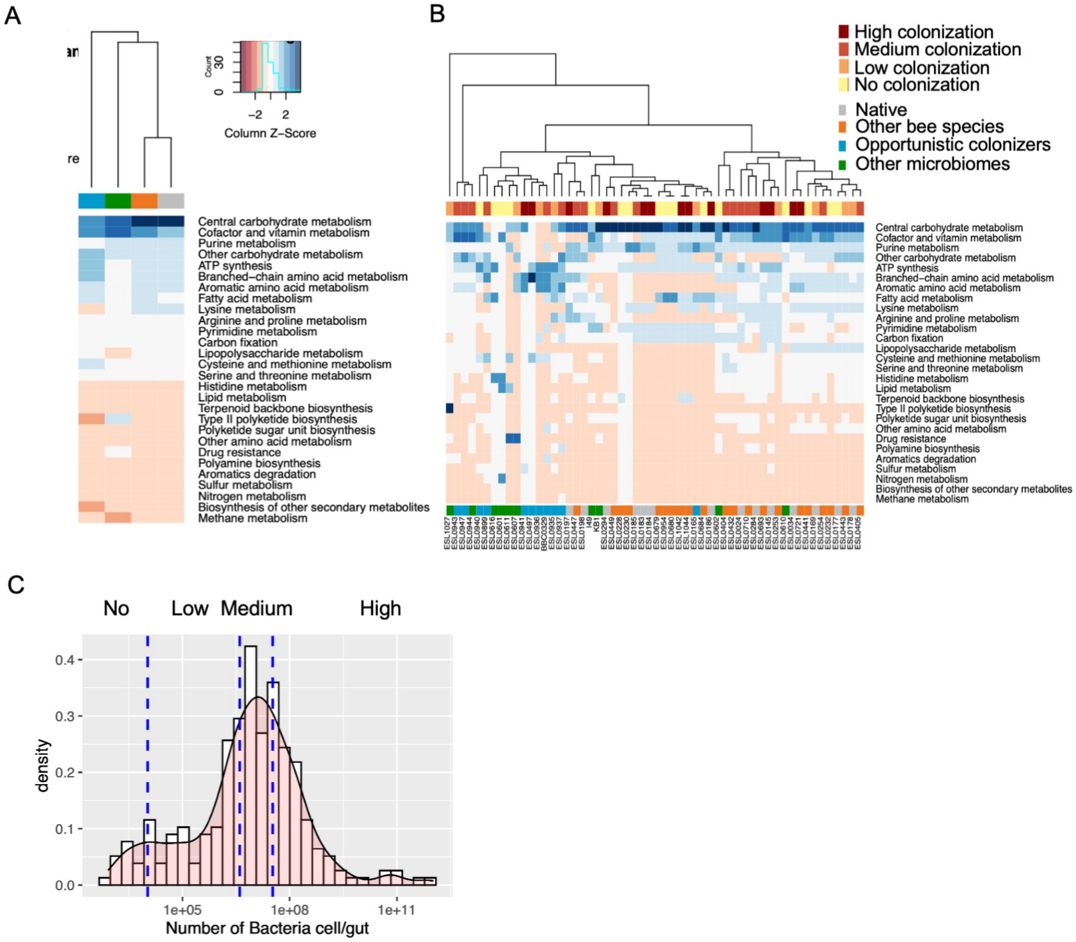
A
B
C
Count
Column Z–Score
High colonization
Medium colonization
Low colonization
No colonization
Native
Other bee species
Opportunistic colonizers
Other microbiomes
Central carbohydrate metabolism
Cofactor and vitamin metabolism
Purine metabolism
Other carbohydrate metabolism
ATP synthesis
Branched−chain amino acid metabolism
Aromatic amino acid metabolism
Fatty acid metabolism
Lysine metabolism
Arginine and proline metabolism
Pyrimidine metabolism
Carbon fixation
Lipopolysaccharide metabolism
Cysteine and methionine metabolism
Serine and threonine metabolism
Histidine metabolism
Lipid metabolism
Terpenoid backbone biosynthesis
Type II polyketide biosynthesis
Polyketide sugar unit biosynthesis
Other amino acid metabolism
Drug resistance
Polyamine biosynthesis
Aromatics degradation
Sulfur metabolism
Nitrogen metabolism
Biosynthesis of other secondary metabolites
Methane metabolism
No
Low
Medium
High
density
Number of Bacteria cell/gut

Figure S6: **A.** The presence and completeness of KEGG modules in the genomes of individual strains categorized by isolation source. Heatmap of % completeness for each strain in each category. Grey: *Native*; orange: *Other bee species*; blue: *Opportunistic colonizers*; green: *Other microbiomes*. **B.** Heatmap of the number of modules that are at least 80% complete for each pathway group. Top label shows bacterial loads categorized in for groups based on the abundance distribution in monocolonization (See materials and methods). **C**. Distribution of bacterial loads in honey bee guts during monocolonization. Strains are categorized based on colonization levels, defined by load percentiles: *No colonization* (below the 25th percentile), *Low colonization* (25th-50th percentile), *Medium colonization* (50th-75th percentile), and *High colonization* (above the 75th percentile).

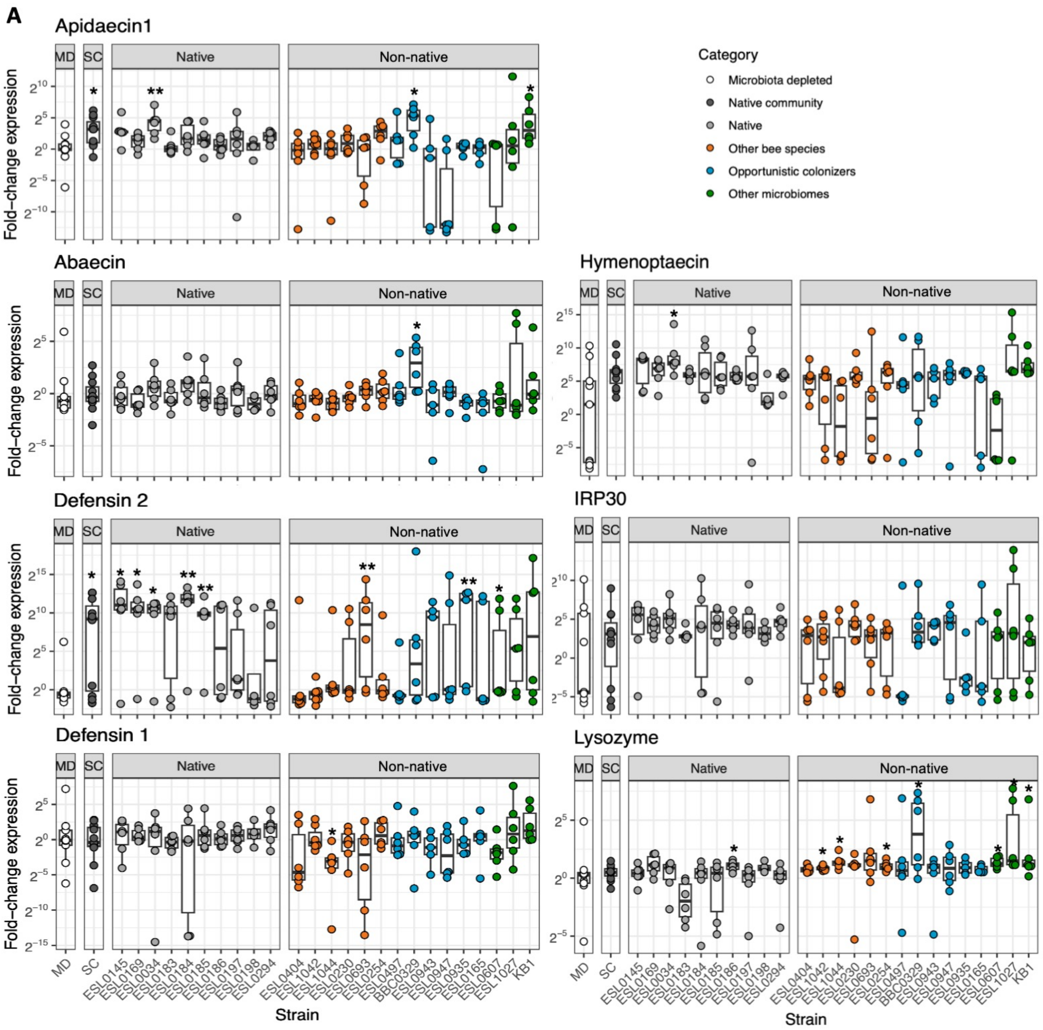

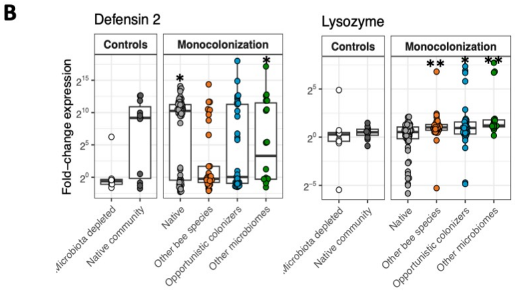

C

Strain	Genus
ESL0145	*Snodgrassella*
ESL0169	*Gilliamella*
ESL0034	*Frischella*
ESL0183	*Lactobacillus*
ESL0184	*Lactobacillus*
ESL0185	*Lactobacillus*
ESL0186	*Lactobacillus*
ESL0197	*Bifidobacterium*
ESL0198	*Bifidobacterium*
ESL0294	*Bombilactobacillus*

Figure S7: **Host antimicrobial peptide responses to colonization by individual bacterial strains. A** Fold-change in expression relative to MD of six antimicrobial peptide (AMP) genes, *Apidaecin1*, *Abaecin*, *Defensin1*, *Defensin2*, *Hymenoptaecin*, *IRP30*, and *Lysozyme,* in the honey bee gut during monocolonization with the indicated bacterial strains. AMP expression was measured relative to microbiota-depleted (MD) bees. Bees colonized with the synthetic native community (SC) are shown as reference controls. Strains are grouped by category and host origin, with separate panels for native and non-native strains. Data are shown as individual points with boxplots indicating the median and interquartile range. Asterisks indicate strains that induced a significant change in expression relative to the MD control (P < 0.05, Wilcoxon rank-sum test, FDR-corrected, n=6). Supplementary File S5. **B,** Summary of *Defensin2* and *Lysozyme* expression levels across different strain categories in control (MD and SC) or monocolonization conditions. Asterisks indicate strains that induced a significant change in expression relative to the MD control (P < 0.05, Wilcoxon rank-sum test, FDR-corrected). No significant differences in expression were observed among categories for *Apidaecin1*, *Abaecin*, *Defensin1*, *Hymenoptaecin*, or *IRP30*. **C,** Genus corresponding to each Strain ID for figure S8.

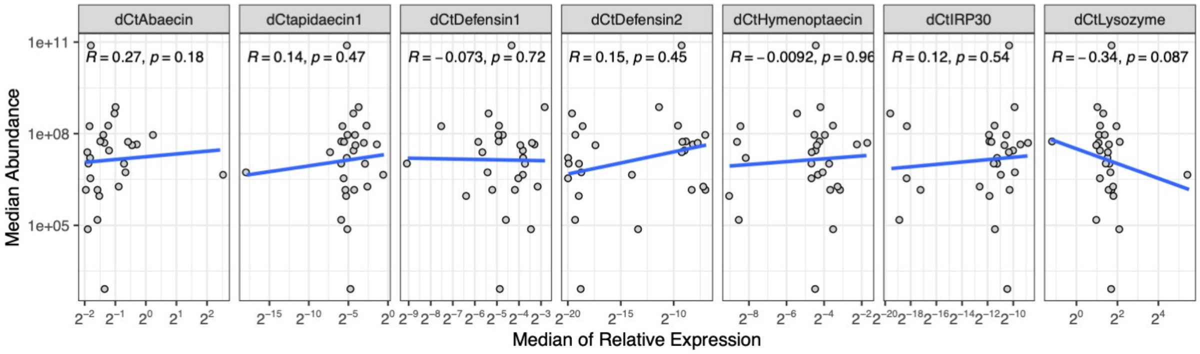

Figure S8: **Antimicrobial peptide expression at seven days post-colonization does not predict bacterial colonization efficiency.** Correlation between antimicrobial peptide (AMP) expression and bacterial colonization abundance in the honey bee gut. Scatter plots show the

relationship between the median relative expression of seven AMP genes (*Abaecin*, *Apidaecin1*, *Defensin1*, *Defensin2*, *Hymenoptaecin*, *IRP30*, and *Lysozyme*) and the median abundance of bacterial strains across samples. Each point represents a bacterial strain. Blue lines indicate linear regression fits. Spearman correlation coefficients (*R*) and associated *P* values are shown for each gene. Regression lines are shown for visualization only; statistical support for associations is assessed using the Spearman correlation coefficients and *P* values reported in each panel.

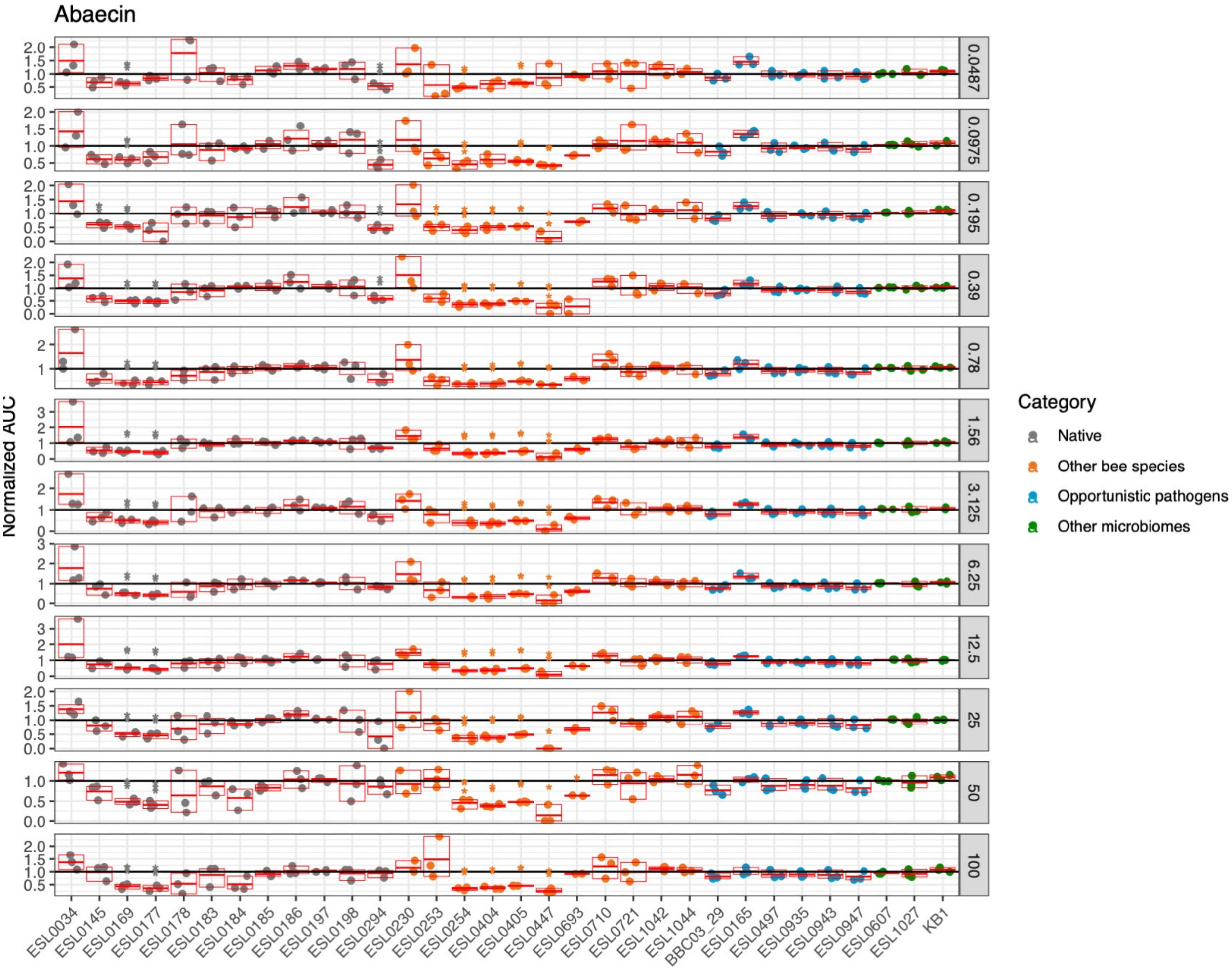

Figure S9. Area under the curve (AUC) of bacterial growth, normalized to the untreated control and presented on a logarithmic scale. The right panel shows the AUC values at a representative

concentration of Abaecin. Each condition was tested in triplicate (*n* = 3). Statistical significance was assessed using a one-sample Wilcoxon test against the null hypothesis of no growth inhibition (normalized value = 1), with *P* values adjusted for multiple comparisons using the false discovery rate (FDR) correction. Significance levels are indicated as follows: ****$P \leq 0.0001$, ***$P \leq 0.001$, **$P \leq 0.01$, *$P \leq 0.05$.

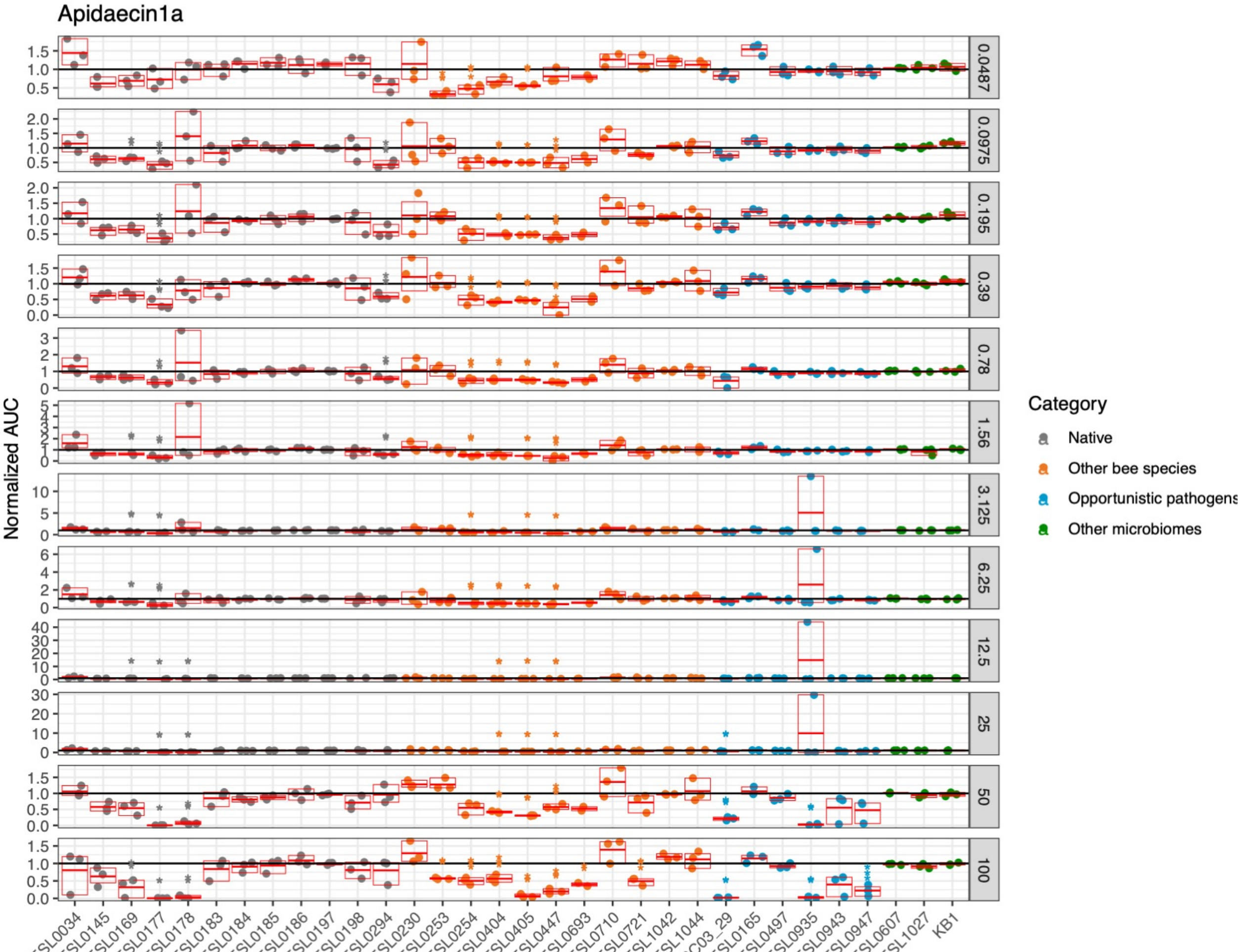

Figure S10. Area under the curve (AUC) of bacterial growth, normalized to the untreated control and presented on a logarithmic scale. The right panel shows the AUC values at a representative concentration of Apidaecin 1a. Each condition was tested in triplicate (*n* = 3). Statistical significance was assessed using a one-sample Wilcoxon test against the null hypothesis of no

growth inhibition (normalized value = 1), with P values adjusted for multiple comparisons using the false discovery rate (FDR) correction. Significance levels are indicated as follows: ****$P \leq 0.0001$, ***$P \leq 0.001$, **$P \leq 0.01$, *$P \leq 0.05$.

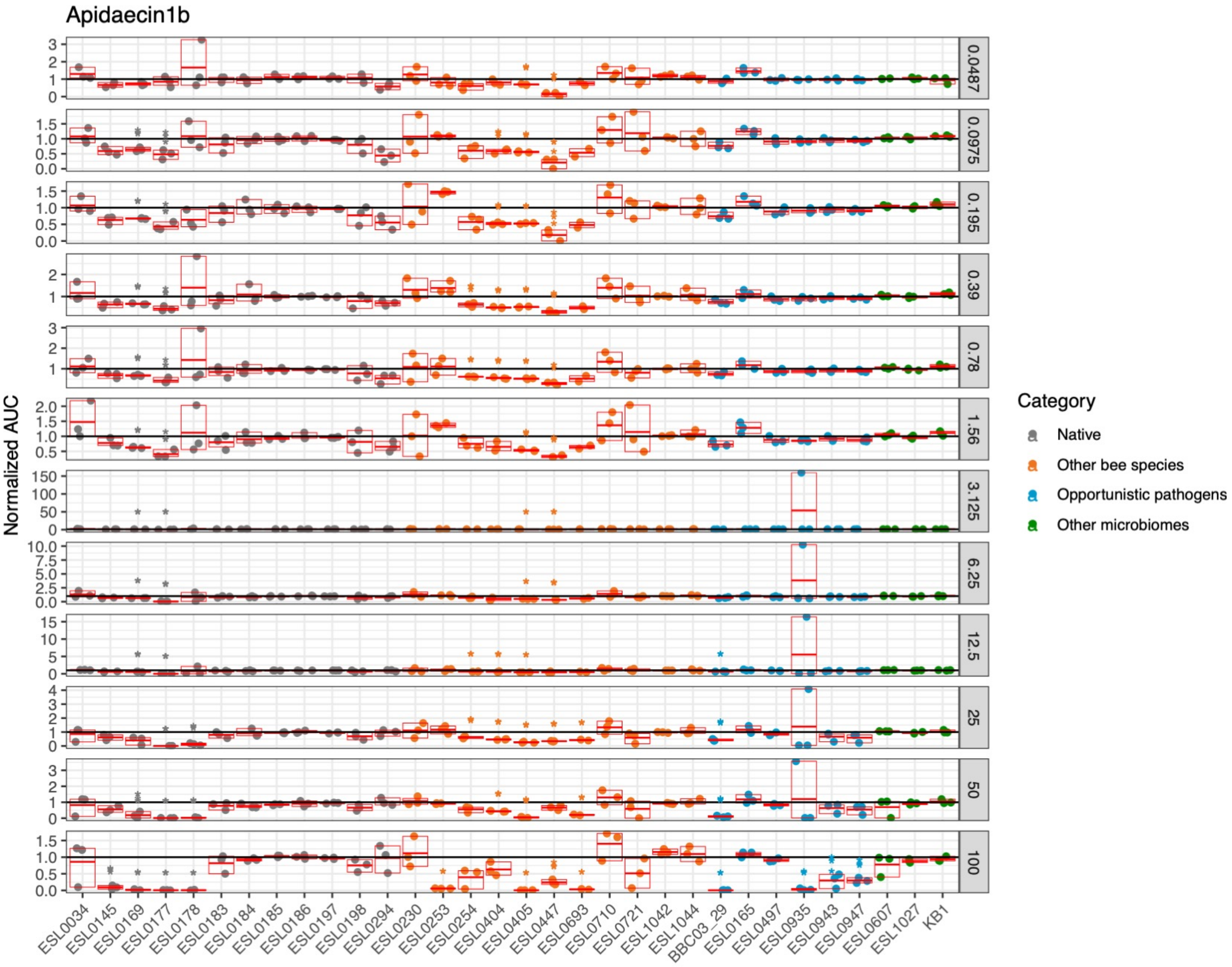

Figure S11. Area under the curve (AUC) of bacterial growth, normalized to the untreated control and presented on a logarithmic scale. The right panel shows the AUC values at a representative concentration of Apidaecin 1b. Each condition was tested in triplicate (n = 3). Statistical significance was assessed using a one-sample Wilcoxon test against the null hypothesis of no

growth inhibition (normalized value = 1), with P values adjusted for multiple comparisons using the false discovery rate (FDR) correction. Significance levels are indicated as follows: ****$P \leq 0.0001$, ***$P \leq 0.001$, **$P \leq 0.01$, *$P \leq 0.05$.

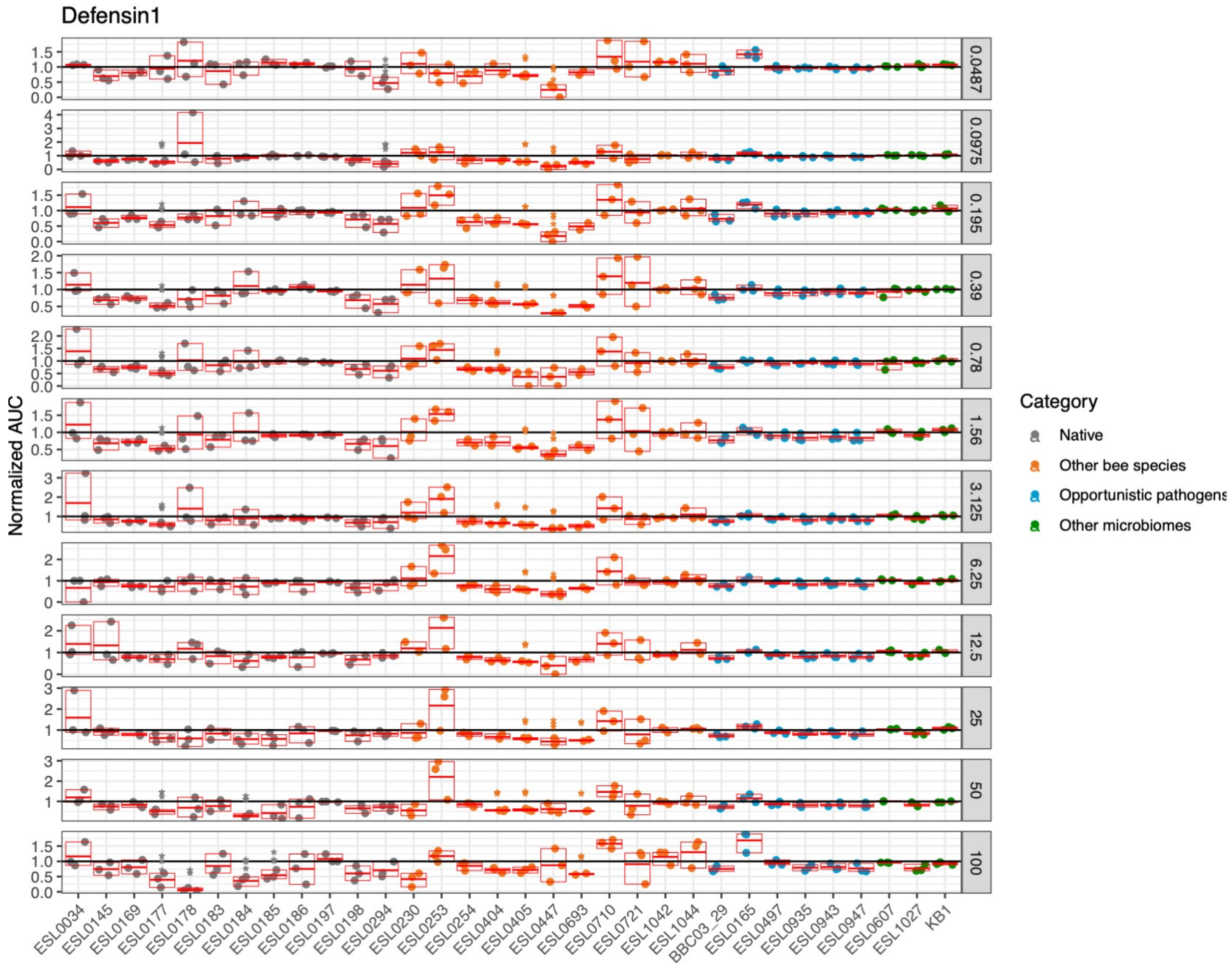

Figure S12. Area under the curve (AUC) of bacterial growth, normalized to the untreated control and presented on a logarithmic scale. The right panel shows the AUC values at a representative concentration of Defensin 1. Each condition was tested in triplicate ($n = 3$). Statistical significance was assessed using a one-sample Wilcoxon test against the null hypothesis of no growth inhibition (normalized value = 1), with P values adjusted for multiple comparisons using the false discovery rate (FDR) correction. Significance levels are indicated as follows:

****$P \leq 0.0001$, ***$P \leq 0.001$, **$P \leq 0.01$, *$P \leq 0.05$.

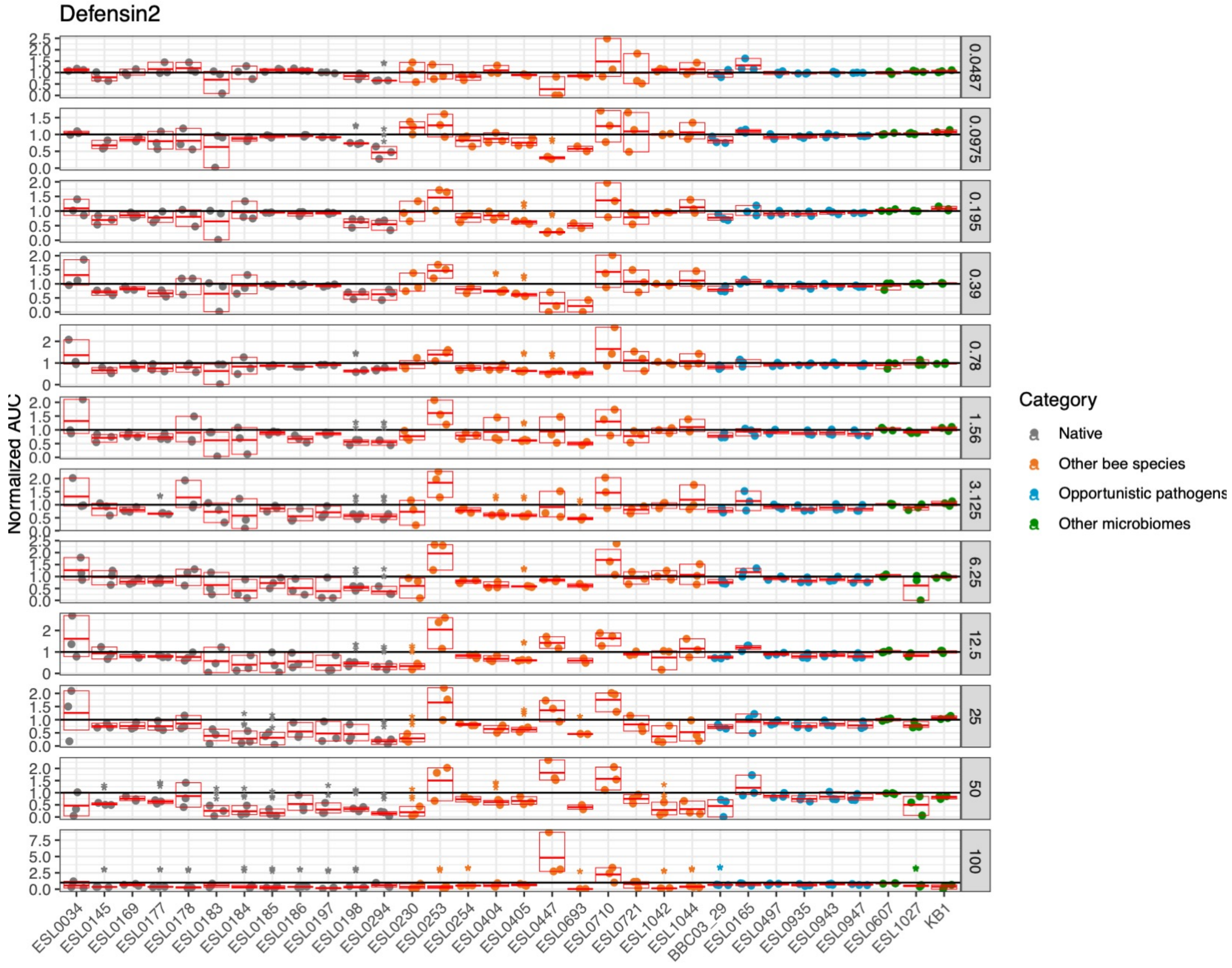

Figure S13. Area under the curve (AUC) of bacterial growth, normalized to the untreated control and presented on a logarithmic scale. The right panel shows the AUC values at a representative concentration of Defensin 2. Each condition was tested in triplicate (*n* = 3). Statistical significance was assessed using a one-sample Wilcoxon test against the null hypothesis of no growth inhibition (normalized value = 1), with *P* values adjusted for multiple comparisons using the false discovery rate (FDR) correction. Significance levels are indicated as follows: ****$P \leq 0.0001$, ***$P \leq 0.001$, **$P \leq 0.01$, *$P \leq 0.05$.

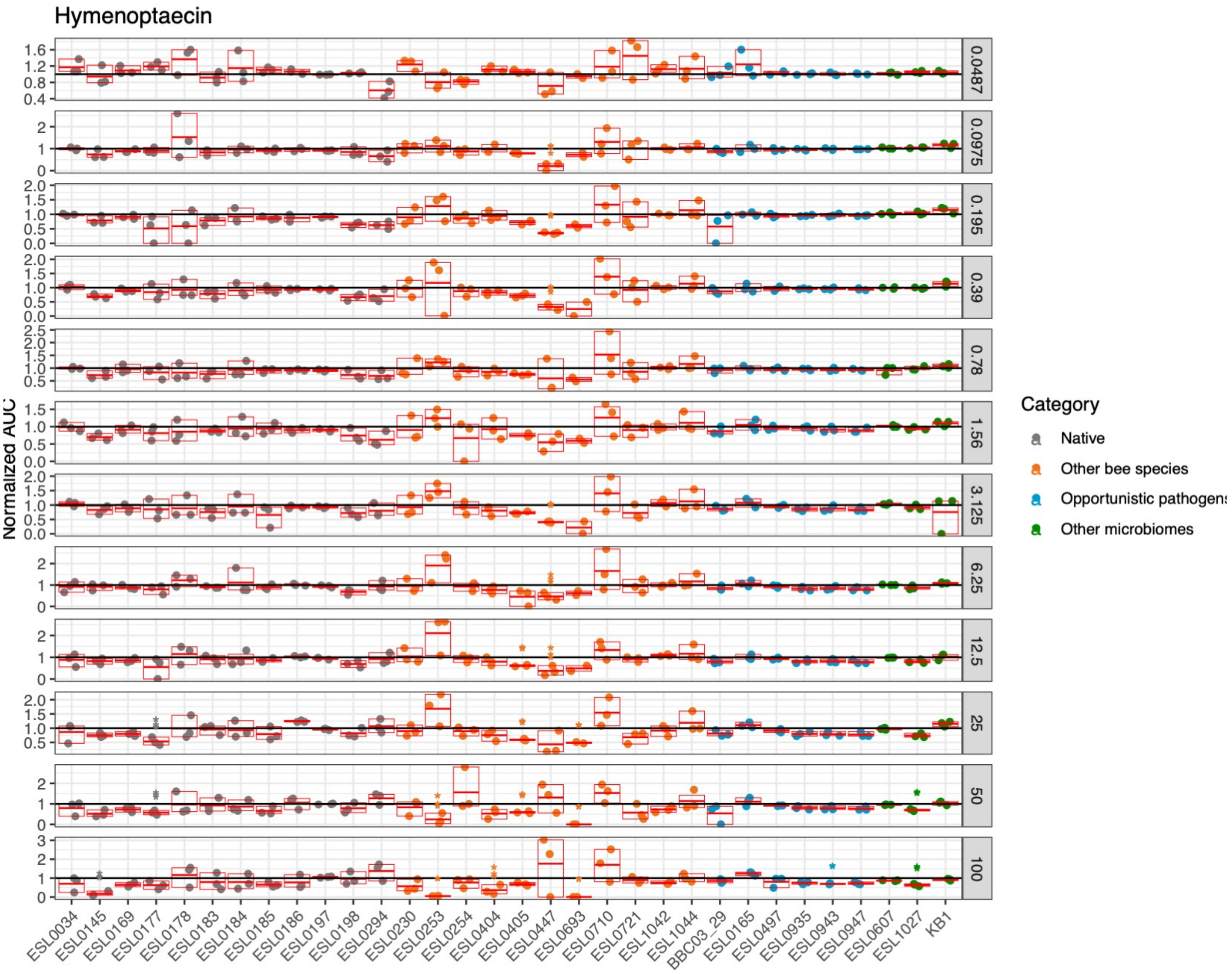

Figure S14. Area under the curve (AUC) of bacterial growth, normalized to the untreated control and presented on a logarithmic scale. The right panel shows the AUC values at a representative concentration of Hymenoptaecin. Each condition was tested in triplicate (*n* = 3). Statistical significance was assessed using a one-sample Wilcoxon test against the null hypothesis of no growth inhibition (normalized value = 1), with *P* values adjusted for multiple comparisons using the false discovery rate (FDR) correction. Significance levels are indicated as follows: ****$P \leq 0.0001$, ***$P \leq 0.001$, **$P \leq 0.01$, *$P \leq 0.05$.

SUPPLEMENTARY FILES

Supplementary File 1: Strain list.

Supplementary File 2: Primer list.

Supplementary File 3: qPCR abundances data.

Supplementary File 4: KEGG modules. Sheet 1: All KEEG modules detected. Sheet 2: Number of modules per strain with a >80% completeness, Sheet 3: Mix-model significant results with abundance data. Sheet 4: Mix-model significant results with percent of colonization data.

Supplementary File 5: qPCR host expression data.

Supplementary File 6: AMP resistance assay. Sheet 1: AMP sequence information. Sheet 2: Growth data parameters. Sheet 3: AMP sensitivity score.

Supplementary File 7: Genome assembly statistics for the bacterial strains sequenced in this study.
